# Comprehensive Analysis of the Immune and Prognostic Implication of TRIM8 in Breast Cancer

**DOI:** 10.3389/fgene.2022.835540

**Published:** 2022-03-17

**Authors:** Cheng Yan, Qingling Liu, Mingkun Nie, Wei Hu, Ruoling Jia

**Affiliations:** ^1^ School of Pharmacy, Xinxiang University, Xinxiang, China; ^2^ Key Laboratory of Nano-carbon Modified Film Technology of Henan Province, Xinxiang University, Xinxiang, China; ^3^ Diagnostic Laboratory of Animal Diseases, Xinxiang University, Xinxiang, China; ^4^ School of Physical Education, Xinxiang University, Xinxiang, China; ^5^ Xinyang Sericulture Test Station, Xinyang, China

**Keywords:** TRIM8, breast cancer, prognosis, immunomicroenviroment, nomogram

## Abstract

**Background:** Breast cancer remains one of most lethal illnesses and the most common malignancies among women, making it important to discover novel biomarkers and therapeutic targets for the disease. Immunotherapy has become a promising therapeutic tool for breast cancer. The role of TRIM8 in breast cancer has rarely been reported.

**Method:** Here we identified TRIM8 expression and its potential function on survival in patients with breast cancer using TCGA (The cancer genome atlas), GEO (Gene expression omnibus) database and METABRIC (Molecular Taxonomy of Breast Cancer International Consortium). Then, TIMER and TISIDB databases were used to investigate the correlations between TRIM8 mRNA levels and immune characteristics. Using stepwise cox regression, we established an immune prognostic signature based on five differentially expression immune-related genes (DE-IRGs). Finally, a nomogram, accompanied by a calibration curve was proposed to predict 1-, 3-, and 5-year survival for breast cancer patients.

**Results:** We found that TRIM8 expression was dramatically lower in breast cancer tissues in comparison with normal tissues. Lower TRIM8 expression was related with worse prognosis in breast cancer. TIMER and TISIDB analysis showed that there were strong correlations between TRIM8 expression and immune characteristics. The receiver operating characteristic (ROC) curve confirmed the good performance in survival prediction and showed good accuracy of the immune prognostic signature. We demonstrated the model usefulness of predictions by nomogram and calibration curves. Our findings indicated that TRIM8 might be a potential link between progression and prognosis survival of breast cancer.

**Conclusion:** This is a comprehensive study to reveal that tripartite motif 8 (TRIM8) may serve as a potential prognostic biomarker associating with immune characteristics and provide a novel therapeutic target for the treatment of breast cancer.

## Introduction

Breast cancer is the most common malignancy among women, which accounts for about one-third of female cancers (Bray et al., 2004; Ginsburg et al., 2017). With the evolution of novel treatment strategies including neoadjuvant treatment, advances in surgical resection, chemotherapy, radiotherapy, and targeted therapy, the survival rates for women with invasive breast cancer are 90% at 5 years, and 84% at 10 years, respectively (Soerjomataram et al., 2008; Silva et al., 2012). Although survival has improved over time, large inequalities remain. The survival rate at 5 years of breast cancer patients is 90.2% in the United States, 89.5% in Australia, and 83.2% in China, but only 66.2% in India and 65% in Malaysia (Allemani et al., 2018). In addition, the incidence of breast cancer rates continues to increase by approximately 0.5% per year (Sung et al., 2021). Previous studies showed that approximately 20–30% of early breast stage cancer would develop into metastatic disease and about 6–10% of female breast cancer patients had stage IV disease at diagnosis. The 5-year survival rate of those patients with distant metastases dropped to 27% (Wang et al., 2019; Siegel et al., 2020). Thus, breast cancer is still a global problem of reduction in breast cancer mortality, particularly for patients with advanced breast cancer (Autier and Boniol, 2018). Age, stage, tumor grade, tumor type, and lymphovascular status as prognostic factors have been widely used in the routine diagnosis and treatment of breast cancer (Gajdos et al., 1999). However, accuracy of these prognostic factors can be limited for predicting overall survival and progression-free survival. Novel biomarkers and molecular signature are urgently needed.

Recently, accumulating evidence shows that the immune system plays an active role in carcinogenesis and progression of cancer (Gonzalez et al., 2018). Immunotherapy has become a promising therapeutic tool for various cancers, such as breast cancer, lung cancer, and malignant melanoma (Botticelli et al., 2020). Several studies have shown that tumor-infiltrating lymphocytes (TILs) in cancer tissue demonstrates to be correlated with favorable prognosis in various malignancies, especially breast cancer (Nanda et al., 2016; Loi et al., 2019; Botticelli et al., 2020). Moreover, immune checkpoint inhibitors (ICIs) dramatically improve the survival of patients with advanced malignancies in several solid tumors (Darvin et al., 2018). These findings have successfully ignited interest in immune-based strategies for breast cancer treatment and prevention.

TRIM8 (Tripartite motif 8) belongs to the tripartite motif protein family consisting of 551 residues, which ubiquitinates various substrates as an E3 ubiquitin ligase (Marzano et al., 2021). Contrasting data highlight its role either as an oncogene or as a tumor suppressor gene, acting as a “double-edged sword” (Liu et al., 2017; Roy et al., 2018; Venuto et al., 2019). Mechanisms underlying TRIM8 regulating cell growth is linked to at least three pivotal cellular signaling pathways: the p53 tumor suppressor, NF-κB, and JAK-STAT pathways. Moreover, recent studies on TRIM8 strongly indicate for the critical role of TRIM8 in immunity and inflammation (Maarifi et al., 2019; Bai et al., 2020). However, it is still unknown the relationship between TRIM8 and immune characteristics in breast cancer.

In this study, we uncovered the immune implication and prognostic impact of TRIM8 in breast cancer. First, we downloaded data from TCGA (The cancer genome atlas), GEO (Gene expression omnibus) database and METABRIC (Molecular Taxonomy of Breast Cancer International Consortium) to evaluate the association between TRIM8 expression and the survival of breast cancer. Then, the correlation of TRIM8 mRNA levels with immune characteristics was systematically investigated. Furthermore, we constructed an immune prognostic signature using differentially expression immune-related genes (DE-IRGs), and validated in the METABRIC database. Finally, a nomogram integrating age, TNM stage, and risk score was constructed to effectively predict individualized survival. Our findings uncovered the potential role of TRIM8 in breast cancer and would move us closer to understand the underlying mechanisms between TRIM8 and tumor-immune.

## Methods and Materials

### Analysis of TRIM8 Expression

Oncomine database (https://www.oncomine. org/resource/login.html), which is an online tumor microarray database, was utilized to analyze the transcription levels of TRIM8 in human cancers (Rhodes et al., 2007). The expression fold change of TRIM8 in different cancers was obtained as the threshold of *p*-value<1E-4, fold change>2 and gene ranking in the top 10%.

Information of TRIM8 expression and survival data in different cancers was obtained from The Cancer Genome Atlas (TCGA, http://www.cancergenome. nih.gov). Using Perl strawberry, we merged the gene expression data of TRIM8 and survival data and extracted the expression amount of TRIM8 across 33 cancer types. The TCGA pan-cancer analysis was performed using R language with a filter threshold that normal sample sizes should be greater than five. Then, we performed expression analysis of TRIM8 in breast cancer between normal tissues (n = 113) and tumor tissues (n = 1,104) using R language. Finally, the expression of TRIM8 in breast cancer was observed by Immunohistochemistry (IHC) images using the Human Protein Atlas (THPA) (https://www.proteinatlas.org/).

### Survival Analysis

TCGA breast cancer dataset was obtained from UCSC Xena browser (http://xena.ucsc.edu/) which included 1,104 cancer samples and 113 non-cancerous samples. Microarray dataset GSE7390, GSE21422, GSE29431, and GSE42568 were obtained from GEO database. METABRIC was obtained from cbioportal, which contained 2,509 primary breast cancer tissues with 548 matched normals. Patients in each of these three sets were separated into high-expression and low-expression groups using the minimum *p*-value approach. Next, we employed Kaplan-Meier plots to perform overall survival (OS), progression-free survival (PFS) probabilities and relapse free survival (RFS) using R ‘‘survival’’ package and R “survminer” package. At last, the R “Meta” package was used to meta-analyze on these three datasets.

### Identification of Differentially Expression Genes and Enrichment Analysis

Differentially expression genes (DEGs) between high-expression (n = 790) and low-expression (n = 292) groups based on TCGA cohort was carried out by the Mann Whitney WilcoxonTest. The screening standard of differential genes were as follows: FDR< 0.05 and |log2 FC| >1. Then, Gene Ontology (GO) and Kyoto Encyclopedia of Genes and Genomes (KEGG) biological process enrichment of differential genes was performed by R software including packages of “clusterProfiler”, “org.Hs.eg.db”, “enrichplot”, “ggplot2”, and “GOplot”. A *p* value < 0.05 was considered statistically significant.

### Associations Between TRIM8 and Immune Characteristics

Tumor Immune Estimation Resource (TIMER) (https://cistrome.dfci.harvard.edu/TIMER/) is web tool that provides a comprehensive resource for analysis of molecular characterization of tumor-immune interactions in diverse cancer types. By taking advantage of this web tool, we studied the relationship between copy number variations of TRIM8 and six immune infiltrates (B cells, CD4^+^ T cells, CD8^+^ T cells, neutrophils, macrophages, and dendritic cells) in breast cancer.

TISIDB (http://cis.hku.hk/TISIDB/index.php) is a web portal for investigation of tumor-immune interactions, which collects multiple types of cancer datasets from the TCGA database. It was used to analyze TRIM8 expression in different immune characteristics, including tumor-infiltrating immune cells and immune immunomodulators.

### Identification of Immune Related Genes

A total of 2,483 immune genes were obtained from immport (https://www.immport.org/home). Then, the intersection of these immune genes and 192 DEGs were selected as DE-IRGs.

### Identification of Prognostic Genes Signatures

We used the samples from TCGA cohort as a training set. Univariate Cox regression analysis was employed to determine the DE-IRGs with prognostic significance using R “survival” package. Genes with *p* < 0.05 were screened for further analysis. Later, we further performed multivariate Cox regression to obtain the key DE-IRGs related to the prognosis of breast cancer patients.

### Construction and Validation of the Prognostic Model

After the key DE-IRGs were chosen, the risk score equation was generated as follow:

$$\mathrm{Risk}\;\mathrm{score}=\beta_{\mathrm{1X1}}+\beta_{\mathrm{2X2}}+\cdot \cdot \cdot +\beta_{\mathrm{iXi}}.$$

In this formula, x_i_ was the expression value of the gene, while β_i_ was the risk coefficient of the genes obtained from multivariate Cox regression. The patients were divided into the high-risk group and low-risk group according to the median risk score. The R “survival” package was used to perform the overall survival analysis and obtain Kaplan-Meier survival plots. To evaluate the predictive accuracy of the prognostic model, we conducted ROC curves analysis by R “timeROC” package. Simultaneously, we used samples from the METABRIC database as the validation set. The formula obtained from the training set was also used to assess breast cancer patients in a validation cohort. The Kaplan-Meier analysis was used to assess the predictive ability of DE-IRGs.

### The Construction of Nomogram and Calibration Curves

A nomogram and calibration plots were built by the “rms” package in R software. The nomograms were generated to predict 1, 3, and 5-year survival rates in breast cancer patients. Calibration curves were established to visualize the discriminations between actual 1, 3, and 5-year survival rates and predict overall survival.

## Results

### The Expression Level of TRIM8 in Human Cancers

**FIGURE 1:**
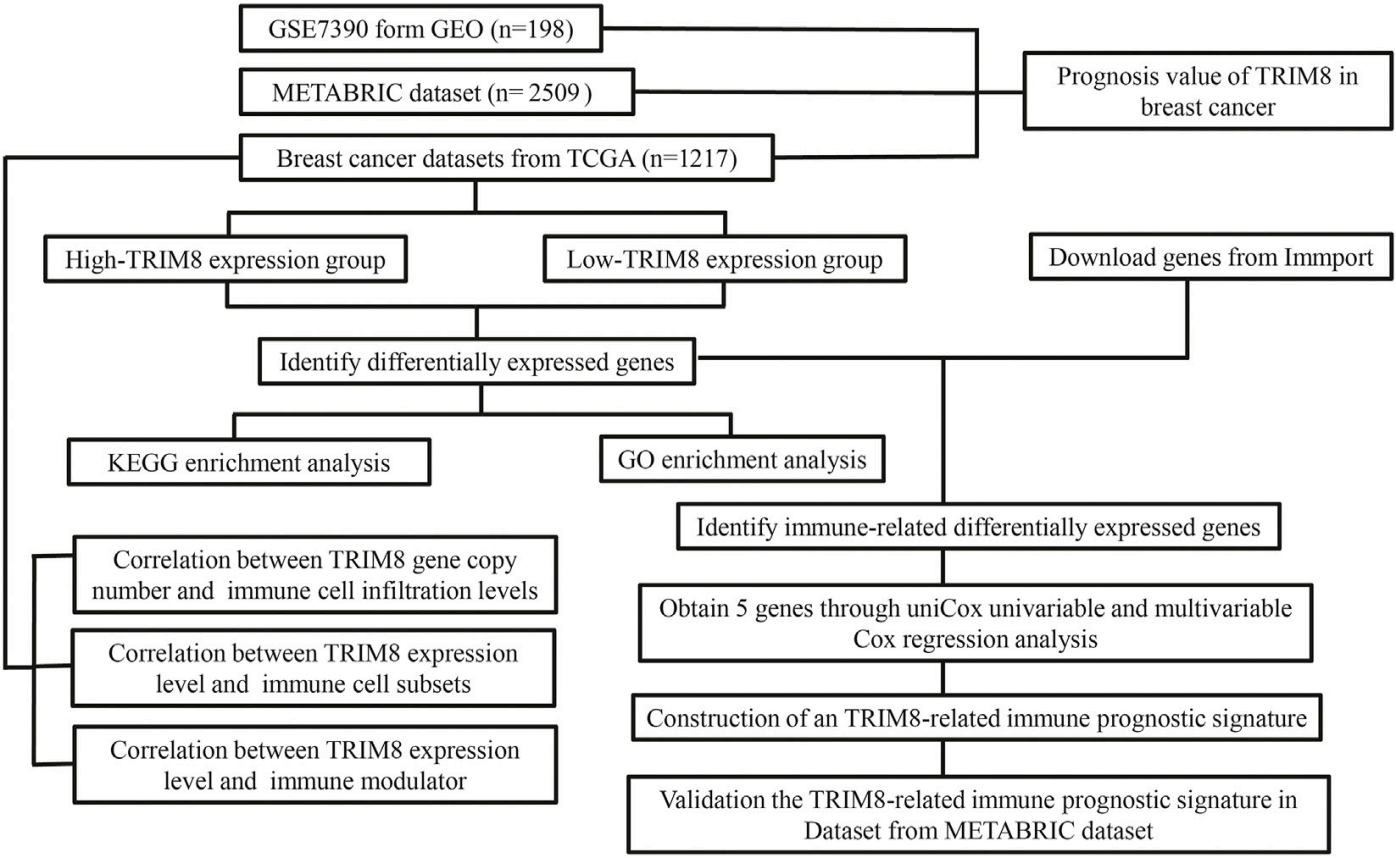
Flowchart of the major steps in this study.

**FIGURE 2:**
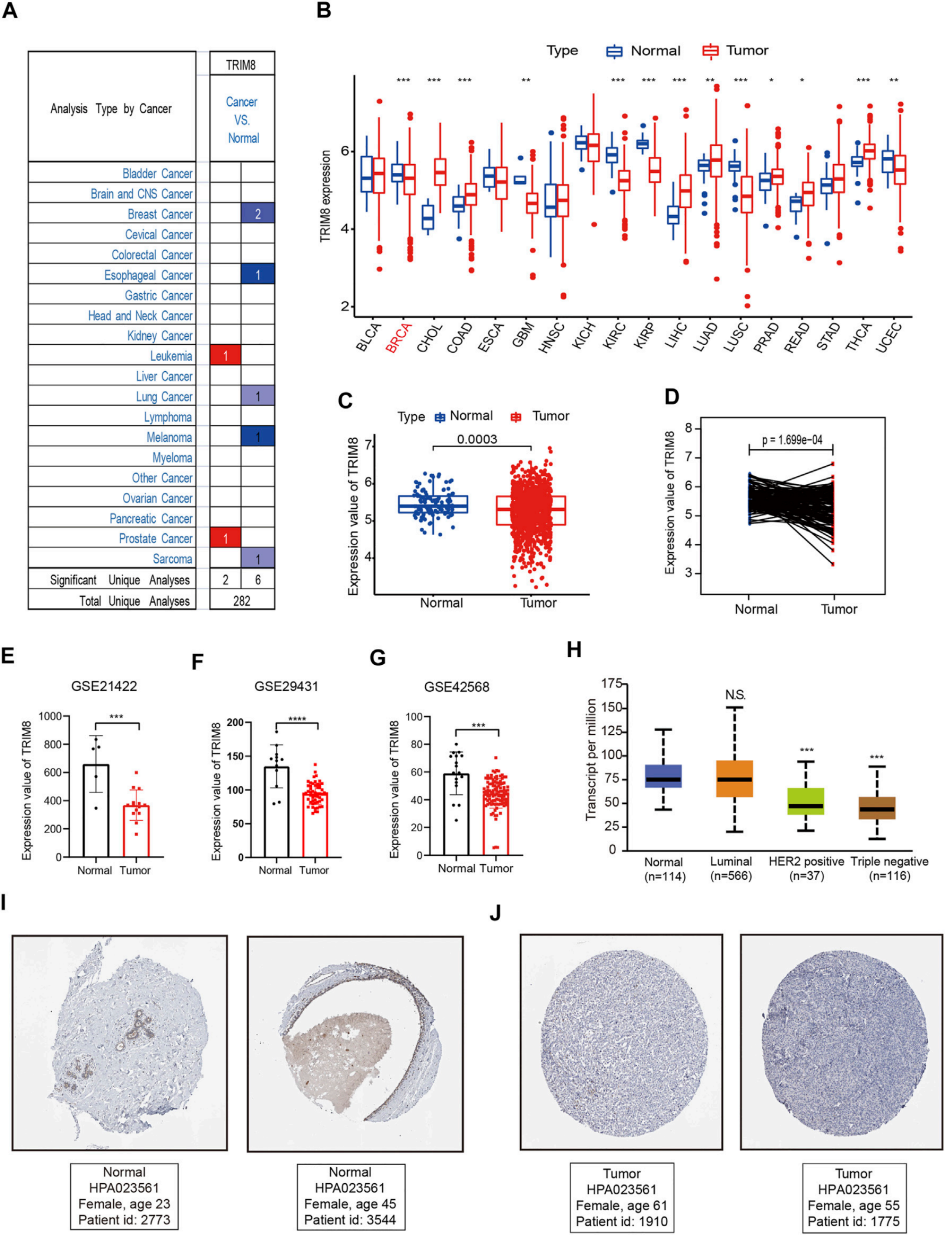
TRIM8 expression levels in different types of human cancers **(A)** The expression level of TRIM8 in different cancer tissues compared with normal tissues from the Oncomine database. Numbers in red and blue represented dataset numbers in which levels of TRIM8 were statistically increased or decreased. **(B)** TRIM8 expression levels in different cancer tissues compared with normal tissues from breast cancer and normal tissues from TCGA database. Red dots represented tumor and blue represented normal **(C)** TRIM8 expression levels of normal and breast cancer tissue in TCGA. **(D)** Relative expressions of TRIM8 mRNA in 224 pairs of breast cancer tissues and adjacent noncancerous tissues from TCGA database **(E)** TRIM8 expression levels of normal and breast cancer tissues in GSE21422. **(F)** TRIM8 expression levels of normal and breast cancer tissues in GSE29431 **(G)** TRIM8 expression levels of normal and breast cancer tissues in GSE42568. **(H)** Boxplots showed TRIM8 expression levels in different molecular subtypes patients with breast cancer (I–J) The images obtained from The Human Protein Atlas database. Representative IHC images showed TRIM8 expression in normal tissues **(I)** and breast cancer tissues **(J)**.

The overall workflow of this study was shown in Figure 1. To evaluate TRIM8 expression in human cancer, we used Oncomine databases to analyze the differential expression of the mRNA levels in cancer and normal tissues. Specifically, this analysis indicated that TRIM8 expression was higher in leukemia and prostate cancer, but was lower in breast cancer, esophageal cancer, lung cancer, melanoma, and sarcoma (Figure 2A). Moreover, to further investigate the expression of TRIM8 in human cancers, we performed a detailed analysis of RNA-Seq data from TCGA. Compared with normal samples, the mRNA expression of TRIM8 was significantly up-regulated in cholangio carcinoma (CHOL), colon adenocarcinoma (COAD), Liver hepatocellular carcinoma (LIHC), Lung adenocarcinoma (LUAD), Prostate adenocarcinoma (PRAD), Rectum adenocarcinoma (READ), and thyroid carcinoma (THCA), but strongly down-regulated in breast invasive carcinoma (BRCA), kidney renal clear cell carcinoma (KIRC), Glioblastoma multiforme (GBM), kidney renal papillary cell carcinoma (KIRP), lung squamous cell carcinoma (LUSC), Uterine Corpus Endometrial Carcinoma (UCEC). We then mapped a scatter plot using TCGA dataset which indicated that TRIM8 was significantly lower in breast cancer as compared with normal tissues (Figure 2C). Furthermore, we also evaluated TRIM8 expression in 224 paired breast cancer tissues in TCGA dataset. TRIM8 expression was dramatically lower in breast cancer tissues in comparison with normal tissues (Figure 2D). Afterwards, we checked three additional independent breast adenocarcinoma gene expression profiles (GSE21422, GSE29431, and GSE42568) to identify the expression level of TRIM8 (Figures 2.E,F,G). Consistent with our previous result, we observed dramatically lower TRIM8 expression in tumor samples compared to normal samples in all of three cohorts. We further evaluated the expression of TRIM8 in different molecular subtype patients with breast cancer in TCGA cohort. Statistically significant differences were observed in HER2^+^ and triple negative breast cancers compared with normal breast tissues (Figure 2H). Finally, Based on the Human Protein Atlas database, the protein expression levels of TRIM8 were evaluated by the HPA023561 antibody. Among 12 breast cancer tissues examined, all of the cases were negative by immunohistochemistry for TRIM8. Representative IHC image showed that TRIM8 staining was higher in breast cancer than in normal tissues (Figure 2,I,J). Taken together, these findings suggested TRIM8 expression in breast cancer samples is lower compared to normal samples.

### Prognostic Value of TRIM8 in Breast Cancer

**FIGURE 3:**
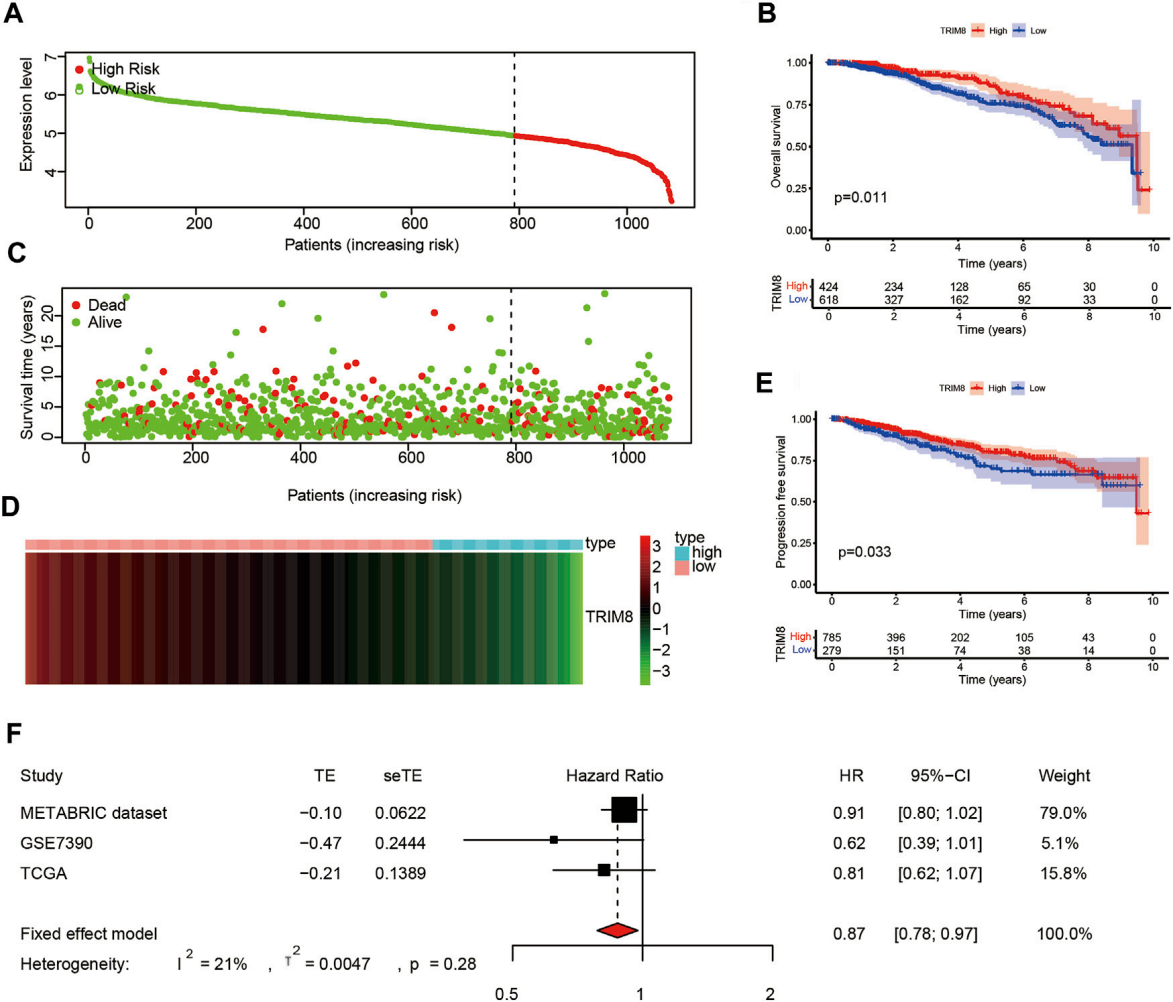
The prognostic value of TRIM8 expression level in breast cancer based on TCGA database **(A)** Patients with breast cancer stratified into high-expression (low-risk; green line) and low-expression groups (high-risk; red line) using the minimum *p*-value approach **(B)** Kaplan‒Meier survival curves showed the over survival probability between the high-risk groups and low-risk groups. **(C)** Survival time and survival status of patients with breast cancer compared between high-risk groups and low-risk groups (setting 10-years as a cut-off). Green plots for alive, red plots for dead **(D)** Heatmap showed the expression of TRIM8 in different groups. Blue color represented high-risk group, while pink color represented low-risk group. **(E)** Kaplan–Meier survival curves displayed progression-free survival (PFS) between the high-risk groups and low-risk groups (setting 10-years as a cut-off). **(F)** Forest plot of high TRIM8 expression with better over survival in breast cancer patients from three databases.

To explore the correlation between TRIM8 expression and prognosis of breast cancer patients, we performed survival analysis using the TCGA database. Based on expression levels, patients were divided into high-expression (*n* = 790) and low-expression (*n* = 292) groups using the minimum *p*-value approach (Figure 3A), and patients with lower expression were more likely to have death (Figure 3C). A heatmap was used to describe the expression of TRIM8 in different groups (Figure 3D). Survival curves were estimated by the Kaplan-Meier analysis which suggested that patients with high TRIM8 expression significantly correlated to longer OS in breast cancer patients (*p* < 0.05) (Figure 3B). Next, we used the Kaplan-Meier analysis to evaluate the correlation between TRIM8 expression levels and PFS (Figure 3E). The result indicated that patients with high TRIM8 expression had a high probability to live (*p* < 0.05). The prognostic value of TRIM8 was then validated with GEO and METABRIC dataset (Supplementary Figure S1). The results were consistent with the results of TCGA. Lastly, we performed a meta-analysis of the three combined datasets to evaluate the prognostic value of TRIM8. The results showed that high TRIM8 expression levels were strongly correlated with longer OS in the breast cancer population (pooled HR = 0.87, 95% CI, 0.78–0.97) (Figure 3F).

### Identification of Differentially Expressed Genes

To better understand the difference between high-expression and low-expression groups, we performed a differentially expression analysis based on TCGA cohort. A total of 192 DEGs were obtained with the cut off criteria of FDR< 0.05 and |log2 FC| >1. The volcano map (Supplementary Figure S2A) showed that 146 DEGs were high-expression, while 46 DEGs were low-expression between high-expression and low-expression groups. Meanwhile, KEGG and GO enrichment analyses were conducted to identify the biological function of 192 DEGs. We found that these 192 DEGs were highly related to Coronavirus disease-COVID-19, complement and coagulation cascades, and IL-17 signaling pathway (Supplementary Figure S2B). Moreover, the results of GO analysis showed the DEGs were mainly concentrated in mammary gland epithelium development, gland morphogenesis, and prostate gland morphogenesis (Supplementary Figure S2C).

### Relationship Between TRIM8 Expression and Immune Cells

**FIGURE 4:**
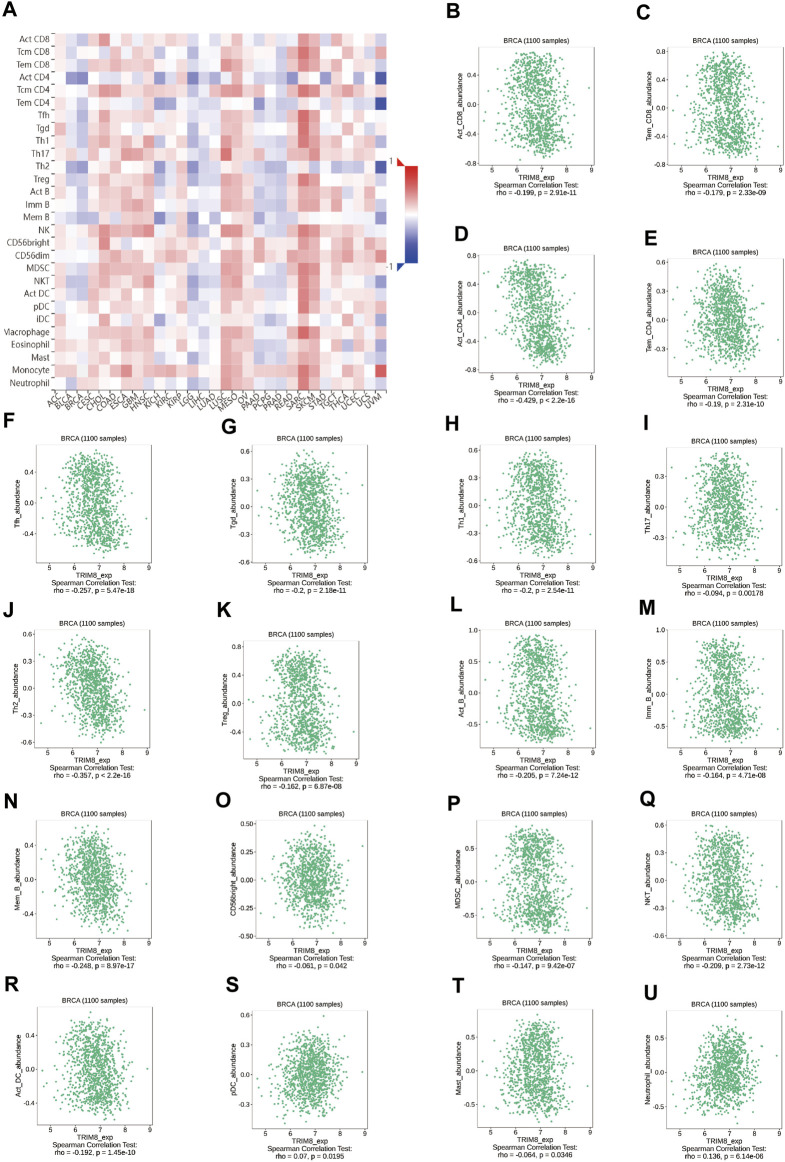
Correlation between the expression of TRIM8 and immune cell subsets in patients with breast cancer based on the TISIDB database **(A)** Heatmap showed the relations between expression of TRIM8 and 28 types of TILs in breast cancer. The red color in the heatmap indicated a positive correlation, the blue color indicated a negative correlation, and the white color indicated a non-significant correlation. **(B–U)** The significantly correlation between the expression of TRIM8 and immune cell infiltrations.

We studied the influence of TRIM8 expression upon the immune system. Initially, we evaluated the correlation between the expression levels of TRIM8 and immune cell infiltration. There were strong correlations between TRIM8 copy numbers and immune cell infiltration, such as macrophage in breast cancer, CD4^+^ T Cell, and Macrophage in luminal-type breast cancer (Supplementary Figure S3). Subsequently, we examined the relationship between TRIM8 expression and TILs using TISIDB database. A close relationship between TRIM8 expression and abundance of 28 TILs in different types of cancer was shown (Figure 4A). Moreover, there was a significant association between TRIM8 expression and abundance of 20 TILs in breast cancer (Figure 4B–U).

### Correlations Between TRIM8 Expression and Immunomodulators

To further investigate the relationship of TRIM8 expression with immunomodulators, we first analyzed the association between the expression of TRIM8 and immuneinhibitors across human cancers. The result showed that the expression levels of TRIM8 was significantly associated with immunoinhibitors in a variety of cancers (Supplementary Figure S4A). Significant association was found between TRIM8 expression and immunoinhibitors in breast cancer (Supplementary Figure S4B–S). Next, we performed correlation analysis of TRIM8 expression with immunostimulators and MHC molecule. The results demonstrated that TRIM8 expression correlated well with parts of immunostimulators and MHC molecule in breast cancer (Supplementary Figure S5, S6).

### Construction of a Prognostic Signature of Breast Cancer in the Train Set

**FIGURE 5:**
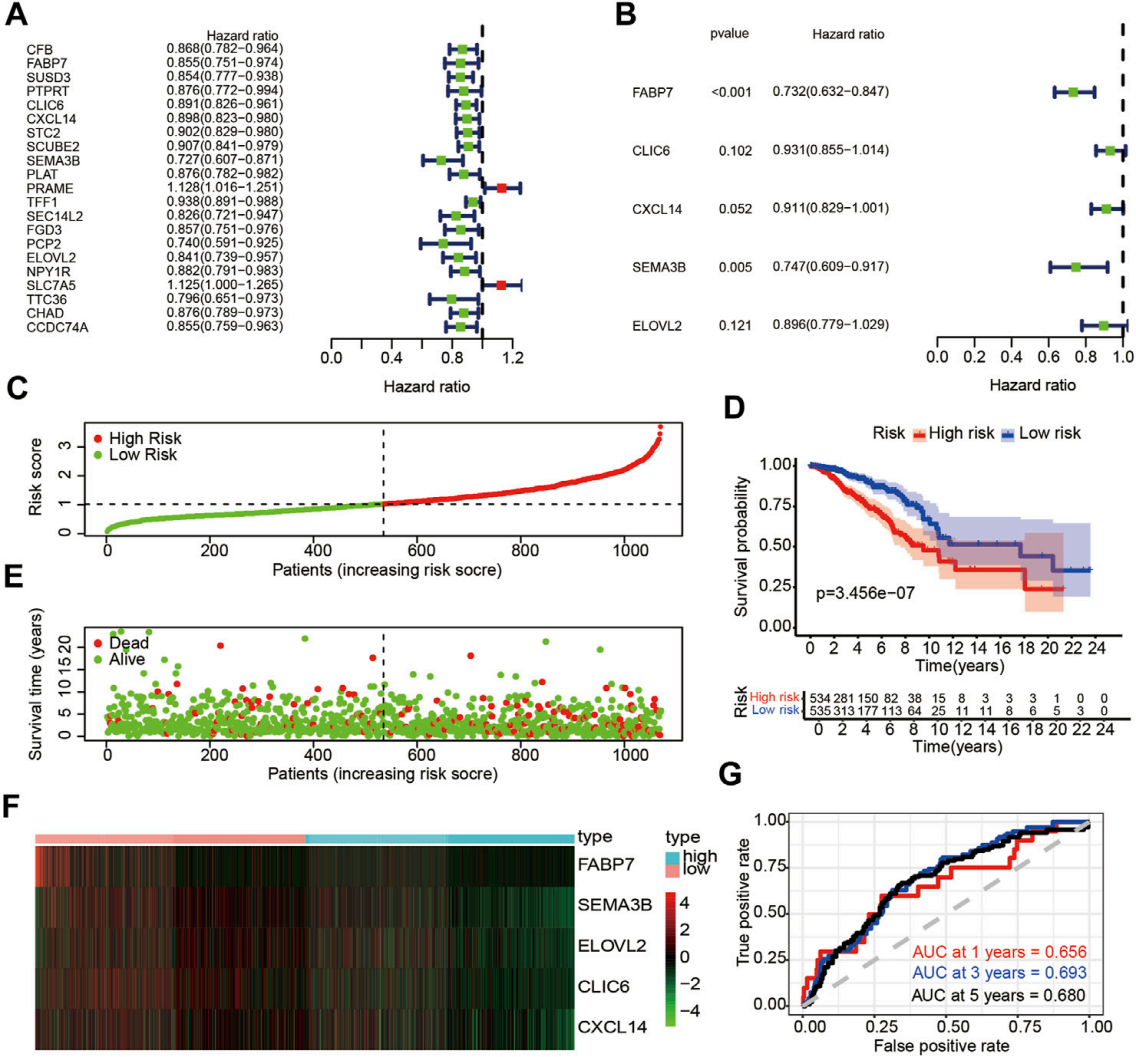
Identification of DE-IRGs with prognostic value in breast cancer **(A)** Univariate Cox regression analysis of DE-ARGs and immune genes. **(B)** Multivariate Cox regression of DE-IRGs based on univariate Cox regression analysis **(C)** The risk score stratified the breast cancer patients into high-risk groups (“High” red line) and low-risk groups (“Low” green line) in training set **(D)** Kaplan-Meier survival curves compared survival probability between the high-risk groups and low-risk group in training set. **(E)** Survival time and survival status of patients in breast cancer compared between high-risk groups and low-risk groups in training set. Green plots for alive, red plots for dead **(F)** Heatmap indicated expression of the five DE-IRGs in breast cancer. Blue color represents high group, while pink color represents low group. **(G)** Time-dependent ROC curves for DE-IRGs model to predict patient survival in training set.

Previous research has demonstrated that TRIM8 expression level is closely related with immunity in breast cancer. Therefore, we sought to determine whether DEGs was related to immune genes in breast cancer. To begin with, we collected 2,483 humanimmune genes from Immport. Finally, there were 23 DE-IRGs within all the immune genes when intersecting with 192 DEGs. In order to confirm the prognostic significance of these immune-related genes in breast cancer, univariate Cox regression analysis was carried out. We screened out 21 immune-related genes which included nineteen potential protective genes and two potential risky genes (Figure 5A). We then pursued multivariate Cox regression analysis and 5 immune-related genes were obtained (Figure 5B). Finally, an immune prognostic signature was constructed based on FABP7, CLIC6, CXCL14, SEMA3B and ELOVL2. Next, we calculated risk score of each patient using the following formulate: risk score= (-0.31212341×FABP7)+(-0.071409412×CLIC6)+(-0.093656818×CXCL14)+(-0.291305101×SEMA3B)+(-0.110268438×ELOVL2). Patients were stratified into high-risk (*n* = 534) and low-risk groups (*n* = 535) based on the median values (Figure 5C). Patients with higher risk scores were more likely than those with lower scores to have death (Figure 5E). The heatmap was used to visualize the differential expression of these five immune-related genes between the two groups (Figure 5F). Survival analysis indicated that patients in the high-risk group had a poorer prognosis than those in the low-risk group (*p* < 0.05) (Figure 5D). As depicted in Figure 5E, the AUC of ROC for 1-year, 3-year and 5-year OS were 0.656,0.693 and 0.680, respectively. Moreover, we investigated relationships between the expression level of these five immune-related genes and OS in breast cancer. The results showed that the higher expression level of these five immune-related genes had a better prognosis than those in the low expression level (Supplementary Figure S7A,C,E,G,I). Together, the above results demonstrated the credibility and effectiveness of the model in predicting the prognosis of patients with breast cancer.

### Validation of the Risk Score of METABRIC Dataset in Test Set

**FIGURE 6:**
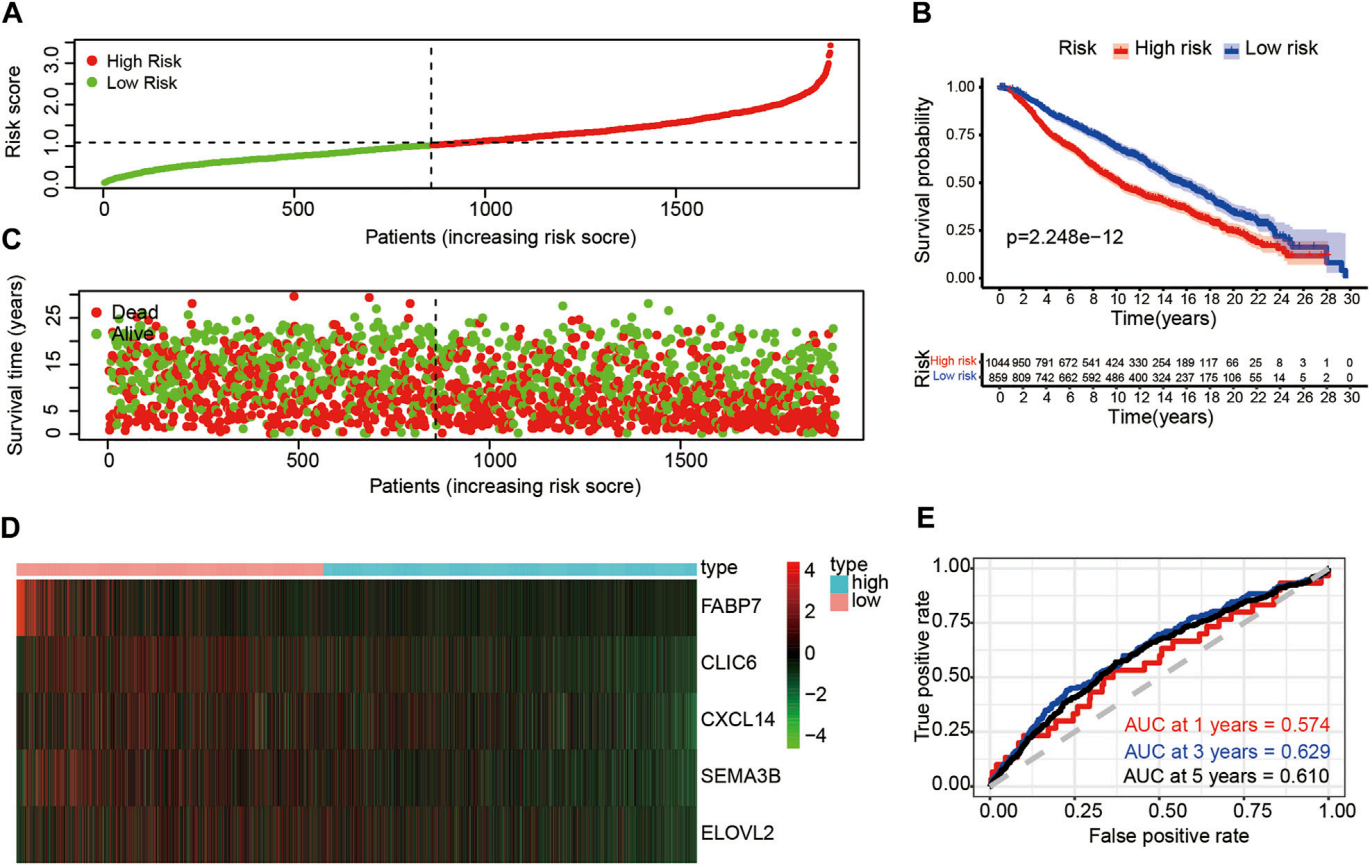
Risk analysis and overall survival of DE-IRGs in the test set **(A)** The risk score stratified the breast cancer patients into high-risk groups (“High” red line) and low-risk groups (“Low” green line) **(B)** Kaplan-Meier survival curves compared survival probability between the high-risk groups and low-risk group. **(C)** Survival time and survival status of patients in breast cancer compared between high-risk groups and low-risk groups. Green plots for alive, red plots for dead **(D)** Heatmap indicated expression of the five DE-IRGs in breast cancer. Blue color represents high group, while pink color represents low group. **(E)** Time-dependent ROC curves for DE-IRGs model to predict patient survival.

To further validate the prognostic value of the risk score, we calculated risk scores for patients in the METABRIC dataset using the same formula of the train set. According to the median value of risk score, the patients were divided into high-risk (*n* = 1,044) and low-risk groups (*n* = 859) (Figure 6A). Those with higher risk scores were more likely to have a poorer prognosis (Figure 6C). A heatmap was presented to visualize the difference expression levels of the five immune-related genes between test groups (Figure 6D). The results in the test set were similar in the train set. Survival analysis indicated that patients in the high-risk group had a poorer prognosis than those in low-risk group (*p* < 0.05) (Figure 6B). Time-dependent ROC analysis showed that the prognostic accuracy of the immune-related genes signature was 0.574 at 1 year, 0.629 at 3 years and 0.610 at 5 years (Figure 6E). Furthermore, high expression level of CLIC6, CXCL14, SEMA3B and ELOVL2 immune-related genes might indicate better prognosis (*p* < 0.05) (Supplementary Figures S7B,D,F,H,J).

### Construction of the Nomogram and Performance Validation

**FIGURE 7:**
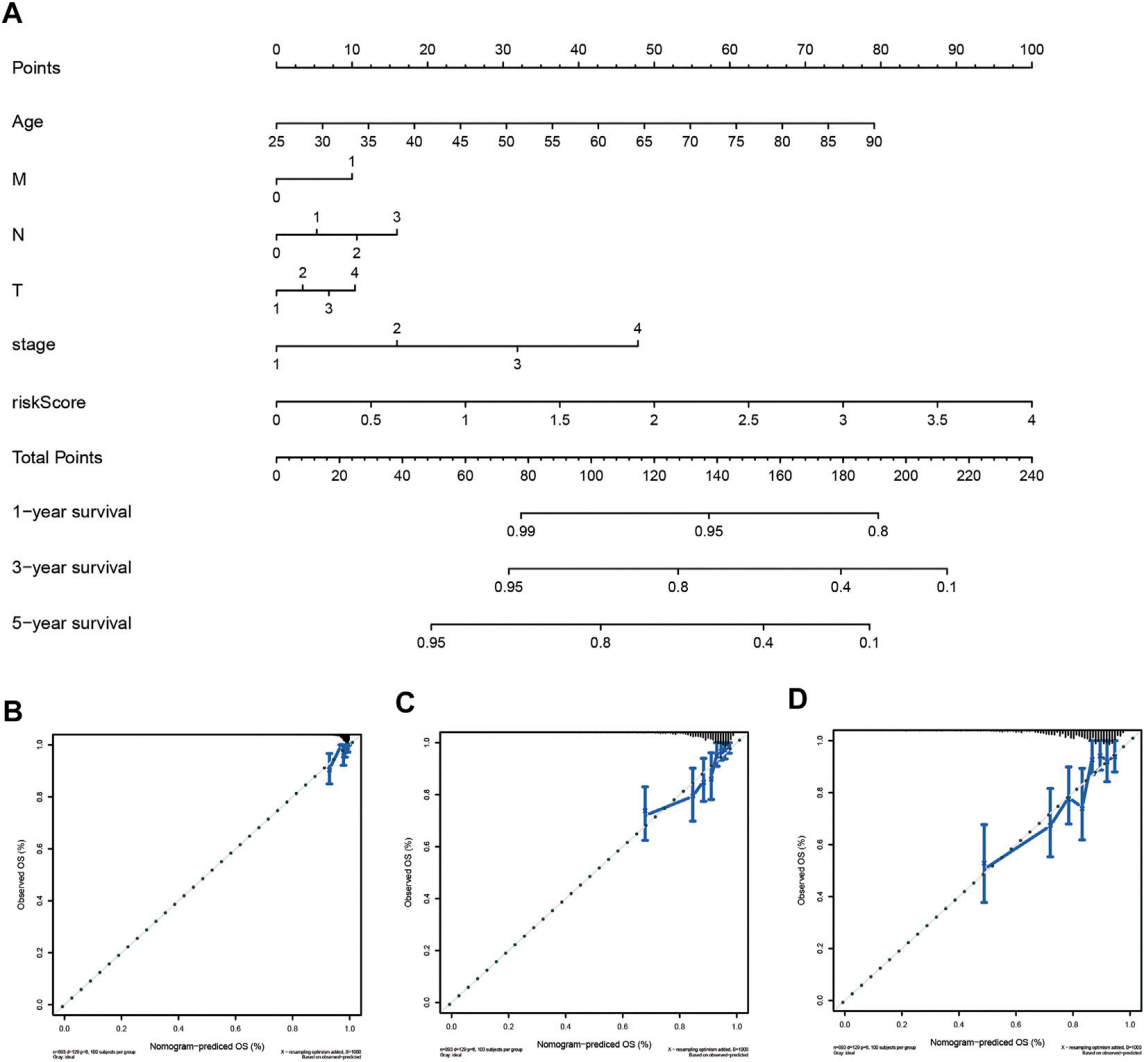
Establishment of the prognostic nomogram to predict overall survival of breast cancer patients based on TCGA cohort **(A)** The nomogram for predicting survival proportion of patients in 1-, 3-, and 5-year. **(B–D)** The calibration plots for predicting patient survival at1-, 3- and 5-years.

To provide the clinician with a better quantitative method to predict prognosis of a breast cancer patient, we established a nomogram with multiple factors including age, M, N, T, stage, and risk score (Figure 7A). The nomogram was used to evaluate the survival probability of 1-, 3-, and 5-years. Nomograms showed a good performance with a high C-index of 0.768, suggesting that it could serve as an effective tool for prognostic evaluation of patients with breast cancer. In addition, we constructed calibration curves which showed that the predicted and actual survival rates were highly in agreement at 1, 3, and 5 years (Figures 7B–D). These findings revealed that the nomogram with our risk scores can improve predicting OS.

## Discussion

Breast cancer, one of the most common malignant tumors among women, seriously threatens female health. Although there have been some advances in early detection, diagnosis, and treatment in recent years, patients with breast cancer in advanced and/or metastatic stage, still have a poor prognosis. Previous studies reveal that the immune system is critical in the development of breast cancer. This sobering data highlight the urgent requirement to identify novel biomarkers and provide promising immune-related therapeutic targets for breast cancer.

In this study, we first determined the expression and prognostic value of TRIM8 in patients with breast cancer. The expression level of TRIM8 was lower in breast cancer tissues than in normal tissues, and high TRIM8 expression levels were strongly correlated with better OS, PFS and RFS in the breast cancer population. Altogether, these findings suggest TRIM8 may serve as a tumor suppressor gene and a putative prognostic biomarker for breast cancer.

With the constant recognition of the importance of tumor immune microenvironment on the disease course of cancers, there is a great potential to predict and guide immunotherapeutic responsiveness (Binnewies et al., 2018). Our results revealed that there is a close linkage between TRIM8 and the immunity of breast cancer. First, we found that TRIM8 gene copy numbers were inversely associated with the infiltration levels in breast cancer, including B cell, CD4^+^Tcell, and macrophage. Then, TISIDB analysis was conducted to study the correlation between TRIM8 expression and immune cells, and we found that there were negative correlations between TRIM8 expression and abundances of CD4^+^T cell, B cell, T follicular helper cell, Gamma delta T cell, Th1 cell, Th2 cell, and natural killer T cell in breast cancer, especially CD4^+^ T cell. Altogether, this evidence indicates a potential role of TRIM8 in immune microenvironment. Early studies showed that immune microenvironment played a vital role in the progression and prognosis of breast cancer (Lu et al., 2020). It was reported that a high expression of CD4^+^ T cells might imply progression of breast cancer and patients with higher expression of CD4^+^ T cell had worse cancer specific OS in breast cancer (Chin et al., 1992; Matkowski et al., 2009). In addition, studies found that high of level Th2 cells were positively correlated with aggressiveness of breast cancer (Zhang et al., 2015; Le et al., 2021), while higher Th2 was associated with a worse prognosis in breast cancer (Teschendorff et al., 2010; Varn et al., 2017; Faucheux et al., 2019). In this study, high expression of TRIM8 indicated a good prognosis in breast cancer. The positive role of TRIM8 expression prognosis of patients with breast cancer was in accordance with different potential functional roles of immune cell types. These findings further indicated that TRIM8 might be a potential link between progression and prognosis survival of breast cancer, which might serve as a biomarker for breast cancer therapies.

At present, immune checkpoint blockade plays a major role in the treatment for cancer therapy, especially for advanced cancers. To better understand the underlying biological function of TRIM8 gene, we conducted TISIDB analysis to determine the correlation between TRIM8 and immunomodulator in breast cancer. The results showed that TRIM8 was significantly positively correlated with PVRL2 but negatively correlated with PD-L1 and the key immunoregulator CTLA-4. PVRL2 was a potential target for cancer immunotherapy which was extensively investigated. Previous studies reported that PVRL2 expression was different in different cancer types. It was highly expressed in acute myeloid leukemia, multiple myeloma, and epithelial cancers. PVRL2 expression was linked to aggressiveness and adverse prognosis in gallbladder cancer. However, other studies demonstrated an opposite role of PVRL2 in hepatocellular carcinoma (HCC), suggesting that patients with high PVRL2 expression on their tumors do better. This view was consistent with our studies. According to our results. TRIM8 might positively regulate the expression of PVRL2 in breast cancer. Notably, our findings might provide a novel molecular target for the treatment of breast cancer.

Immune-related prognostic models have been widely acquired and applied to predict the prognosis of breast cancer patients. Ascierto et al. found that the expression level of immune genes in breast cancer was significantly associated with tumor relapse based on gene expression analysis of breast cancer tissues by microarray, and developed a prognostic gene signature to discriminate the patient subgroup at a significantly increased risk of relapse (Ascierto et al., 2012). Shiyuan Wang et al. established an immune-related prognostic score (IRPS) by combining the normalized enrichment score (NES) values and 54 immune cells, which was significantly correlated with prolonged survival time and several immunogenomic features of breast cancer (Wang et al., 2021). Silei Sui et al. constructed an immune cell infiltration-based immune score model based on resting CD4^+^ T cells, regulatory T cells, gamma-delta T cells, activated NK cells, monocytes, and M0 macrophages to predict the prognosis of breast cancer patients and the effect of chemotherapy (Sui et al., 2020). In this study, we identified 192 DEGs by comparing the two groups based on TRIM8 expression and intersecting with immune genes. Through further Cox regression analysis, a risk score model was established to predict the overall survival of breast cancer based on five immune-related genes. The predictive accuracy of the model was high in both training set and validation set. Then, a nomogram integrating age, TNM stage, and risk score was constructed to effectively predict individualized survival with a C-index of 0.768.

In summary, our findings might offer accurate prognosis prediction patients with breast cancer and provide clinicians with a new reference for breast cancer treatment.

However, this study needs to be expanded in following aspects: first, our study was conducted only based on online databases and to overcome this limitation, we robustly integrated bioinformatic analyses, such as meta-analysis; second, the underlying mechanisms between TRIM8 expression and tumor immunity in breast cancer should be investigated in the future; and finally, the detail mechanisms underlying the prognostic markers remains to be elucidated.

In conclusion, our results suggest that TRIM8 was expressed at low levels in breast cancer tissues, and high expression of TRIM8 in breast cancer patients predicted better prognosis. TRIM8 expression was associated with immune characteristics, such as infiltrating immune cells and immunomodulators. These findings, together suggested that TRIM8, might play a crucial role in the immune microenvironment of breast cancer. Finally, the immune-related prognostic signature, derived from five DE-IRGs, could serve as an independent prognostic biomarker for OS prediction in breast cancer. Therefore, TRIM8 was predicted to be a probable candidate as both a prognostic biomarker and a new immunotherapeutic strategy for breast cancer treatment.

## Data Availability

The original contributions presented in the study are included in the article/Supplementary Material, further inquiries can be directed to the corresponding author.
